# Activation of Human Peripheral Basophils in Response to High IgE Antibody Concentrations without Antigens

**DOI:** 10.3390/ijms20010045

**Published:** 2018-12-22

**Authors:** Yuhki Yanase, Yoshimi Matsuo, Tomoko Kawaguchi, Kaori Ishii, Akio Tanaka, Kazumasa Iwamoto, Shunsuke Takahagi, Michihiro Hide

**Affiliations:** Department of Dermatology, Institute of Biomedical & Health Sciences, Hiroshima University, Hiroshima 734-8551, Japan; yyanase@hiroshima-u.ac.jp (Y.Y.); ymatsuo@hiroshima-u.ac.jp (Y.M.); tomokok@hiroshima-u.ac.jp (T.K.); ishiik@hiroshima-u.ac.jp (K.I.); tantanakiotan@yahoo.co.jp (A.T.); iwamoto2@hiroshima-u.ac.jp (K.I.); takshuns@gmail.com (S.T.)

**Keywords:** basophils, IgE antibody, FcεRI, histamine, CD203c

## Abstract

Basophils and mast cells have high affinity IgE receptors (FcεRI) on their plasma membrane and play important roles in FcεRI-associated allergic diseases, such as pollen allergy, food allergy, chronic spontaneous urticarial (CSU), and atopic dermatitis (AD). To date, several reports have revealed that high IgE antibody concentrations activate mast cells—which reside in tissue—in the absence of any antigens (allergens). However, IgE antibody-induced activation of basophils—which circulate in blood—has not been reported. Here, we investigated whether IgE antibodies may regulate functions of human peripheral basophils without antigens in vitro. We successfully removed IgE antibodies bound to FcεRI on the surface of human peripheral basophils by treating with 0.1% lactic acid. We also demonstrated that high IgE antibody concentrations (>1 μM) induced histamine release, polarization, and CD203c upregulation of IgE antibody-stripped basophils. Thus, high IgE antibody concentrations directly activate basophils, which express IgE-free FcεRI on the cell surface. This mechanism may contribute to the pathogenesis of patients with AD and CSU who have higher serum IgE concentrations compared to healthy donors.

## 1. Introduction

It is well known that basophils circulate in blood and account for less than 1% of peripheral blood leukocytes [1]. Basophils develop from hematopoietic stem cells into mature basophils via granulocyte progenitors in bone marrow and then circulate in blood [2]. Basophils play important roles in antigen- and IgE antibody-associated allergic disorders, such as urticaria, asthma, pollen allergy, food allergen, anaphylactic shock, and atopic dermatitis (AD) [1,3,4]. Basophils express the high-affinity IgE receptors (FcεRI) and IL-3 receptors (IL-3R) on their surface [1,3,4]. Crosslinkage of IgE antibodies bound to FcεRIs by a multivalent antigen (allergen) results in the release of preformed mediators—such as histamine and platelet-activating factor (PAF)—from secretory granules, followed by the generation of newly synthesized mediators including the products of arachidonic acid metabolism and cytokines, such as IL-4 and IL-13 [3,4]. On the other hand, IL-3 and IL-3R interactions play an important role for enhancement of antigen-IgE antibody interaction-induced histamine release, development, and survival of basophil [3,5]. 

Mast cells, resident in tissue, are recognized as similar cells to basophils due to their characters and functions [3,6]. Mast cells also express FcεRIs on their surface and are activated in response to antigen-IgE antibody interactions, resulting in both the release of inflammatory mediators, such as histamines, and morphological changes [6]. To date, monomeric IgE antibody-regulated mast cell functions have been reported by several groups [7,8,9]. Pandey et al. reported that high monomeric IgE concentrations could stimulate rat basophilic leukemia cells (RBL-2H3), resulting in degranulation, membrane ruffling, and NFAT translocation [7]. Kitaura et al. also clarified that high IgE antibody concentrations promote the migration of bone marrow mouse mast cells (BMMCs) [8]. Promotion of mast cell development and modulation of the mast cell phenotype by high monomeric IgE concentrations was reported by Kashiwakura [9]. Thus, high IgE antibody concentrations directly regulate several mast cell functions without binding to antigens.

Therefore, the mechanism of basophils activation induced by antigen-IgE antibody interactions on FcεRIs has been well investigated. However, the mechanism for direct activation of basophils by IgE antibodies themselves has remained unclear. In this study, we investigated if high IgE antibody concentrations directly induce the activation of human peripheral basophils without any antigens.

## 2. Results

### 2.1. Concentration of IgE Antibodies in Serum of Healthy Donors and Patients with Skin Allergic Disorders

The elevation of IgE antibodies in serum of patients with chronic spontaneous urticaria (CSU) and patients with atopic dermatitis (AD) has been reported by several groups in Europe and the USA [10,11]. Here, we measured IgE antibodies in sera of healthy donors, patients with CSU, and patients with AD in Japan. As shown in Figure S1a, the concentrations of IgE antibodies in sera of patients with AD or CSU are significantly higher than those in sera of healthy donors. Moreover, the levels of IgE antibodies in sera are proportional to the level of IgE receptors (FcεRI) expressed on the surface of human peripheral basophils (Figure S1a).

### 2.2. Stripping of IgE Antibodies Bound to FcεRIs on the Surface of Human Peripheral Basophils

**Figure 1:**
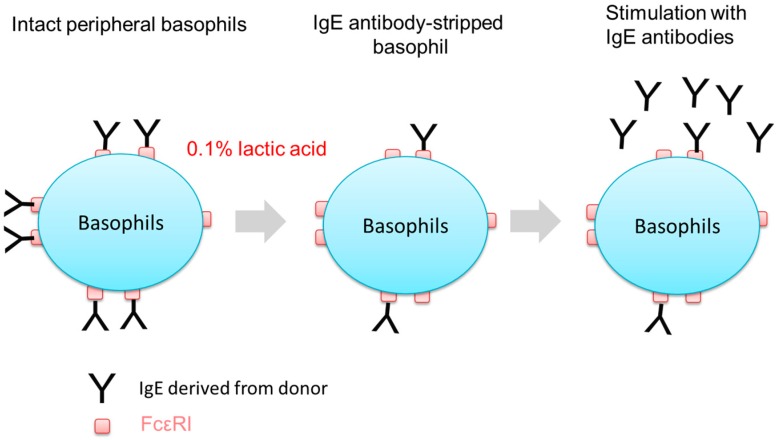
Schematic of removal of IgE antibodies from peripheral blood basophils and activation of IgE antibody-stripped basophils in response to IgE antibodies.

**Figure 2:**
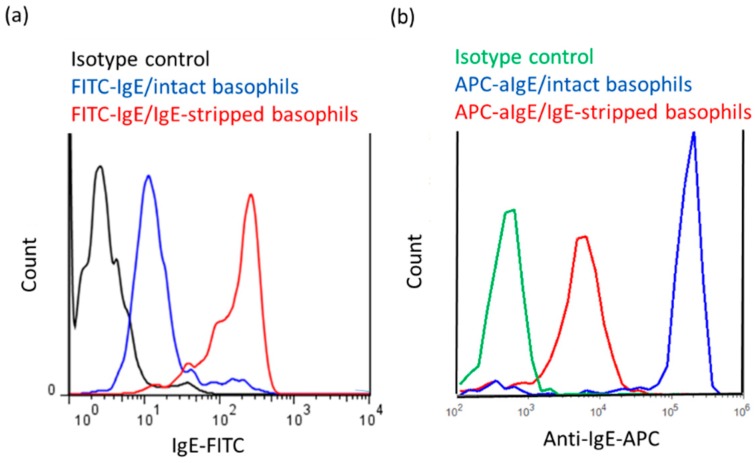
Removal of IgE antibodies on FcεRI and detection of IgE-FITC bound to FcεRIs or anti-IgE-APC bound to IgE antibodies on the surface of peripheral blood basophils. (**a**) Intensity of FITC-labeled IgE antibodies on the surface of basophils treated with or without lactic acid. (**b**) Intensity of APC-labeled anti-IgE antibodies (APC-aIgE) on the surface of basophils treated with or without lactic acid.

Since almost all IgE receptors of basophils isolated from human peripheral blood are already occupied by IgE antibodies circulating in a donor’s blood due to the high affinity of FcεRI for IgE antibodies (Kd around 10^−10^ M) [12], we first tried to remove IgE antibodies on FcεRIs of basophils by treatment with 0.1% lactic acid saline (Figure 1). As shown in Figure 2a, the fluorescent intensity of IgE-FITC bound to FcεRI on the surface of IgE-stripped basophils were clearly increased compared to intact peripheral basophils. Moreover, Figure 2b shows that IgE antibodies on FcεRIs detected by anti-IgE-allophycocyanin (APC) are decreased by treatment with lactic acid, suggesting that treatment with lactic acid successfully removed a large part of IgE antibodies which were originally bound to FcεRIs on human peripheral basophils (Figure 2).

### 2.3. Activation of Human Peripheral Basophils in Response to Human IgE Antibodies

**Figure 3:**
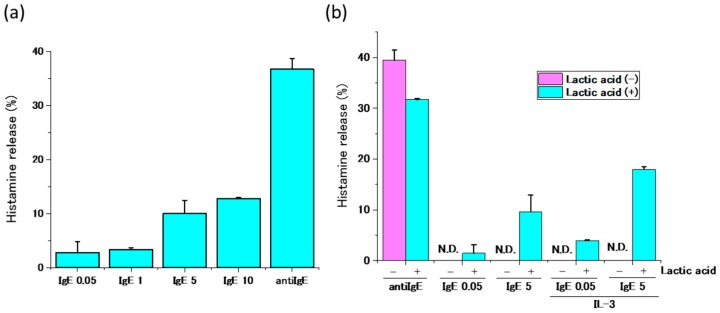
Histamine release from human peripheral basophils in response to high IgE antibody concentrations. (**a**) IgE antibodies (0.05–10 μg/mL) induce histamine release from IgE antibodies-stripped basophils at concentration dependent manner. (**b**) IL-3 (1 ng/mL) enhanced IgE antibodies-induced histamine release. N.D. means “not detected”.

We then investigated the effect of various concentrations of IgE antibodies on the functions of human peripheral basophils of which IgE antibodies on FcεRI were partially removed by the treatment with 0.1% lactic acid saline. Though the low concentrations—50 ng/mL—of IgE antibodies that are usually applied for IgE sensitization for mast cells in vitro could not activate basophils, high IgE antibody concentrations (>1 μg/mL) induced the release of histamines from IgE-stripped basophils at the concentration dependent manner (Figure 3a). High IgE antibody concentrations obtained from other sources (Human IgE (P50) from Nordic-MUbio, Human IgE (HE1) from BioPorto Diagmpstocs A/S) also induce release of histamine from IgE antibody-stripped basophils (data not shown). On the other hand, anti-IgE antibodies induced histamine release from basophils that were not treated with lactic acid saline, and high IgE antibody concentrations did not induce histamine release. These results imply that IgE antibody concentrations greater than 1 μg/mL may activate IgE-stripped basophils in the absence of any antigens. Though IL-3 alone did not induce histamine release from basophils treated with or without lactic acid (data not shown), IgE antibody-induced release of histamine from basophils treated with lactic acid was enhanced in the presence of IL-3 (1 ng/mL) (Figure 3b). 

**Figure 4:**
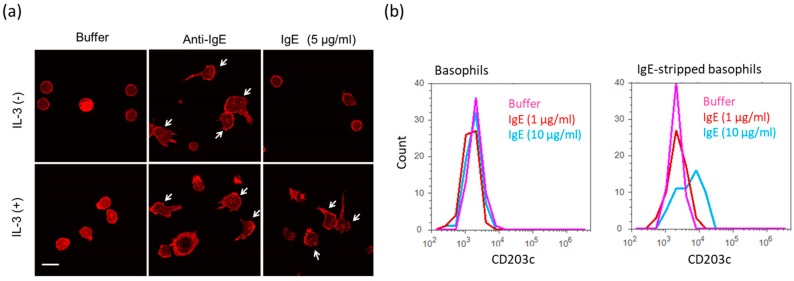
Various activations of human peripheral basophils in response to high IgE antibody concentrations. (**a**) Morphological changes of human basophils on the fibronectin-coated glass slide in response to IgE antibodies (5 μg/mL) with or without IL-3. White arrows indicate polarizing cells. White bar shows ca. 10 μm. (**b**) Expression levels of CD203c on the surface of basophils in response to IgE antibodies (10 μg/mL).

We then investigated the effect of high IgE antibody concentrations on the adhesion and polarization of human peripheral basophils. Though IgE antibodies (5 μg/mL) without IL-3 slightly induced polarization of IgE stripped basophils, high IgE antibody concentrations (5 μg/mL) with IL-3 (1 ng/mL) clearly increased the number of IgE stripped basophils with polarity on the fibronectin coated glass-slide. This suggests that IgE antibodies together with IL-3 induce the migration of basophils (Figure 4a). Moreover, the slight release of IL-4 from basophils in response to IgE antibodies was also enhanced in the presence of IL-3 (data not shown). Finally, we investigated if the expression of the basophil activation marker CD203c, also called ENPP3, which is involved in hydrolysis of extracellular nucleotides, is enhanced in response to high IgE antibody concentrations. As shown in Figure 4b, the treatment with 10 μg/mL IgE antibodies increased the expression level of CD203c on the surface of cells. Thus, high IgE antibody concentrations may regulate functions of human peripheral basophils even in whole blood.

## 3. Discussion

In this study, we demonstrated that high IgE antibody concentrations activate human peripheral basophils functions such as histamine release, polarization, and upregulation of surface antigen (CD203c) in the absence of any antigens (allergens). Histamine release in response to anti-IgE antibodies and analysis of FITC-IgE and APC-anti-IgE bindings by means of flow cytometry revealed that the stripping of IgE antibodies by treatment with 0.1% lactic acid saline did not remove all IgE antibodies bound to FcεRIs on the surface of basophils (Figure 2a,b). However, our results demonstrated that basophils bearing IgE-free FcεRIs may directly be activated by IgE in the absence of any antigens (allergens). Moreover, we clarified that high IgE concentrations-induced activation of basophils are enhanced by physiological concentrations of IL-3 (1 ng/mL), which suggests that the IL-3 is also a co-regulator of IgE antibody-induced activation of human peripheral basophils. Furthermore, similar results were observed not only in basophils from healthy donors, but also in basophils from patients with AD (date not shown).

The critical role of IgE antibodies in the development of allergic disorders, such as chronic urticaria and AD, is supported by the effectiveness of anti-IgE antibodies, such as omalizumab (Xolair^®^), in a number of clinical studies [13,14]. In some patients with AD and CSU, the serum levels of IgE antibodies are elevated over 1 μg/mL (Figure S1a). Since basophils have short life cycles (less than three days), basophils that have matured in the bone marrow and have migrated to peripheral blood may be activated in the blood by high IgE antibody concentrations in minutes. They may then become stable enough to be activated in response to exogenous antigens (Figure S2). On the other hand, peripheral basophils can be sensitized with just IgE antibodies when the concentrations of IgE antibodies in serum are low (less than 1 μg/mL) (Figure S2), and FcεRI on basophils can gradually become occupied by IgE antibodies in serum. Therefore, decreases of IgE antibodies by treatment with anti-IgE antibodies may decrease symptoms by preventing basophils’ activation in response to high IgE antibody concentrations. 

As shown in Figure S1b, basophils of patients with AD and CSU also exhibit a high expression level of FcεRI compared to that of healthy donors. Therefore, basophils from AD or CSU donors may be more sensitive to high IgE antibody concentrations. In fact, the clinical efficacy of omalizumab in CSU is co-related to a reduction of basophil FcεRI expression [13,15]. Further studies on the relationship between reactions of basophils to high IgE antibody concentrations and the expression level of FcεRIs on basophils should enable us to clarify the role of basophils, particularly in IgE antibodies-related allergic disorders such as AD and CSU.

## 4. Materials and Methods

### 4.1. Reagents

Ficoll-Paque Plus was sourced from GE Healthcare Japan Corporation (Tokyo, Japan). EasySep™ Human Basophil Enrichment Kit was sourced from STEMCELL Technologies (Vancouver, Canada). Reverse-phase HPLC was sourced from Shimadzu (Kyoto, Japan). Tetramethylrodamin B isothiocyanate (TRITC)-phalloidin and adenosine were sourced from Sigma-Aldrich Japan (Tokyo, Japan). Human IgE Quantitative ELISA kit was sourced from Bethyl Laboratories, Inc., (Montgomery, TX, USA). Human IgE Purified (AG30P) was sourced from MILLIPORE (Burlington, MA, USA). Human IgE (P50) was sourced from Nordic-MUbio (Susteren, the Netherlands). Human IgE (HE1) was sourced from Bioporto Diagnostics A/S (Hellerup, Denmark). Fluorescein Labeling Kit-NH2 was sourced from Dojindo laboratories (Kumamoto, Japan). Anti-IgE receptor-FITC, anti-CD123-PE, anti-CD203c-APC, anti-IgE-APC, and anti-CD203c-FITC were sourced from BioLegend (San Diego, CA, USA). Allergenicity^®^ Kit was sourced from Beckmann Coulter (Brea, CA, USA). IL-3 was sourced from R&D Systems Inc (Minneapolis, MN, USA).

### 4.2. Isolation of Peripheral Blood Mononuclear Cells (PBMCs) and Basophils from Human Peripheral Blood

Human Peripheral Blood Mononuclear Cells (PBMCs) were isolated from the fresh, heparinized blood of drug-free healthy donors using Ficoll-Paque Plus density gradient separation. Basophils were isolated from PBMCs by magnetic depletion of non-basophils using the EasySep™ Human Basophil Enrichment Kit, as performed in our previous work [16]. Purity of isolated basophils was >90%.

### 4.3. Detection of IgE Antibodies in Serum

Concentrations of IgE antibodies in human serum were measured by ELISA according to the manufacturer’s instructions of Human IgE Quantitative ELISA kit.

### 4.4. Labeling of IgE Antibodies with FITC

FITC was bound to IgE antibodies (MILLIPORE) using Fluorescein Labeling Kit-NH2 according to the manufacturer’s instructions.

### 4.5. Removal of IgEs on the Surface of Basophils

IgE antibodies on the surface of basophils from drug-free healthy donors were removed by the treatment with 0.1% lactic acid (pH 3.9), as described previously [17]. The efficiency of IgE removal from FcεRI of basophils was detected by flow cytometry using FITC-labeled IgE antibodies. 

### 4.6. Detection of IgE-free FcεRI, IgE, Total FcεRI and CD203c on the Surface of Basophils by Means of Flow Cytometry

Expression levels of FcεRI on the surface of basophils were detected by flow cytometry. PBMCs were labeled with anti-FcεRI-FITC, anti-CD123-PE, and anti-CD203c-APC. For the detection of IgE-free FcεRI on the surface of basophils, PBMCs treated with or without 0.1% lactic acid were stained with FITC-labeled IgE antibodies, anti-CD123-PE, and anti-CD203c-APC. For the detection of IgEs on the surface of basophils, PBMCs treated with or without 0.1% lactic acid were stained with anti-CD203c-FITC, anti-CD123-PE, and anti-IgE-APC. Expression levels of CD203c on the surface of basophils were detected by using Allergenicity^®^ kit according to the manufacturer’s instructions. 

### 4.7. Histamine Release Test

Histamine release tests with human basophils were performed as described previously with a goat anti-IgE antibody (670 ng/mL) as positive control [18]. Histamine was extracted and measured by reverse-phase HPLC.

### 4.8. Staining of Actin Cytoskeleton and Observation of Morphology of Human Peripheral Basophils

Human peripheral basophils were stimulated with 10 μg/mL IgE antibodies with or without IL-3 (1 ng/mL) on the fibronectin-coated glass bottom dishes. After 30 min incubation at 37 °C, cells were washed with PBS and fixed with 4% paraformaldehyde. The cells were then treated with PBS containing TRITC-phalloidin for 20 min. Fluorescence was observed using confocal laser scanning fluorescent microscopy.

## 5. Conclusions

We here demonstrated that high IgE antibody concentrations directly activate peripheral basophils, which have IgE-free FcεRIs on the surface of cells. This mechanism may contribute to the pathogenesis of allergic disorders such as AD and CSU.

## Supplementary Materials

Supplementary materials can be found at http://www.mdpi.com/1422-0067/20/1/45/s1.

## Abbreviations

FcεRI	high-affinity IgE receptors
CSU	chronic spontaneous urticaria
AD	atopic dermatitis
PBMC	peripheral blood mononuclear cells
IL	interleukin
IgE	imunoglobulin E
