# Supplementary material for: Activation of Human Peripheral Basophils in Response to High IgE Antibody Concentrations without Antigens
Source: Int J Mol Sci. 2018 Dec 22;20(1):45. doi: 10.3390/ijms20010045 (PMC6337277; doi:10.3390/ijms20010045)
Supplement: Supplementary file 1 [file ijms-20-00045-s001.pdf]

Fig.S1

(a)

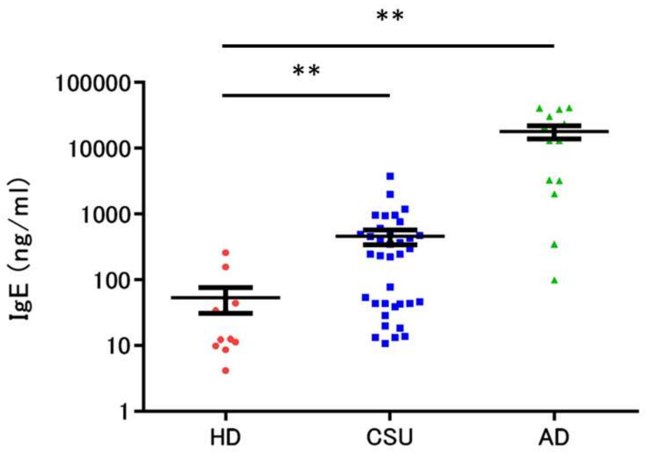

(b)

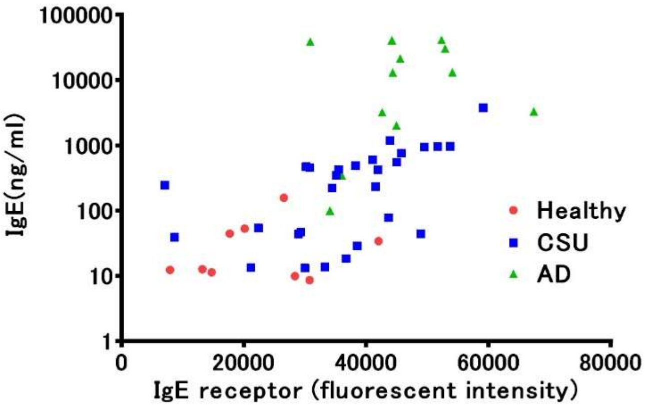

(c)

	Number of patients			Age (mean)
	Male	Female	Total	
HD	5	7	12	34.5
CSU	13	23	36	45.2
AD	7	7	14	30.5

(a) Concentrations of total IgE antibodies in serum of healthy donors (HD), patients with Atopic dermatitis (AD) and chronic spontaneous urticarial (CSU). The differences of the IgE concentrations among healthy donors, CSU donors and AD donors were tested using a one-way ANOVA followed by Tukey's test and considered significant with P<0.01 (**). (b)Relationship between IgE receptors on basophils and IgE antibody concentrations in serum. (c) Subjects information.

Fig.S2

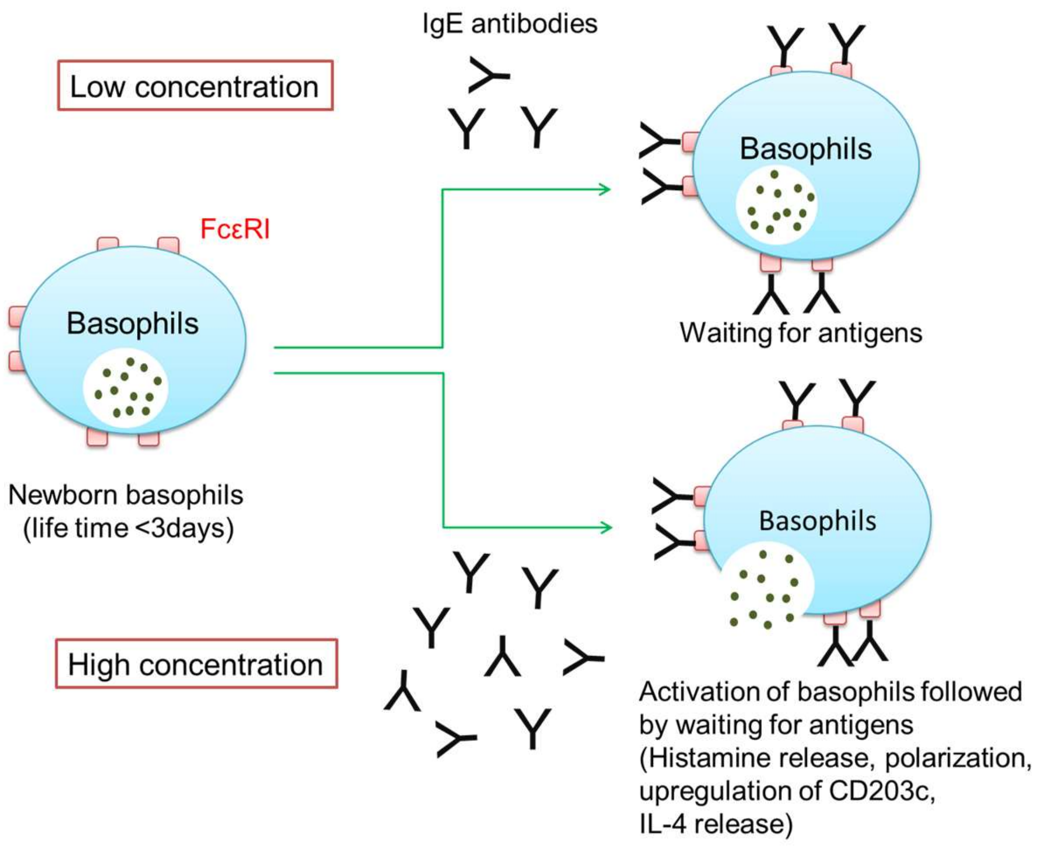

A prospective model of basophils behavior in response to low or high IgE antibody concentrations.
